# Supplementary material for: Certification of non-classicality in all links of a photonic star network without assuming quantum mechanics
Source: Nat Commun. 2023 Apr 14;14:2153. doi: 10.1038/s41467-023-37842-w (PMC10104853; doi:10.1038/s41467-023-37842-w)
Supplement: Supplementary file 1 — Supplementary Information [file 41467_2023_37842_MOESM1_ESM.pdf]

Supplementary Information for: "Certification of non-classicality in all links of a photonic star network without assuming quantum mechanics"

Ning-Ning Wang,[1,2,3,*] Alejandro Pozas-Kerstjens,[4,*,†] Chao Zhang,[1,2,3,†]
Bi-Heng Liu,[1,2,3] Yun-Feng Huang,[1,2,3,†] Chuan-Feng Li,[1,2,3,†]
Guang-Can Guo,[1,2,3] Nicolas Gisin,[5,6] Armin Tavakoli[7,8,†]

[1]CAS Key Laboratory of Quantum Information,
University of Science and Technology of China, Hefei, 230026, China
[2]CAS Center For Excellence in Quantum Information and Quantum Physics,
University of Science and Technology of China, Hefei, 230026, China
[3]Hefei National Laboratory,
University of Science and Technology of China, Hefei, 230088, China
[4]Institute for Mathematical Sciences - ICMAT
(CSIC-UAM-UC3M-UCM), 28049 Madrid, Spain
[5]Group of Applied Physics, University of Geneva, 1211 Geneva 4, Switzerland
[6]Constructor University, Geneva, Switzerland
[7]Physics Department, Lund University, Box 118, 22100 Lund, Sweden
[8]Institute for Quantum Optics and Quantum Information - IQOQI Vienna
Austrian Academy of Sciences, Boltzmanngasse 3, 1090 Vienna, Austria
[*]These authors contributed equally: Ning-Ning Wang and Alejandro Pozas-Kerstjens.
[†]Corresponding authors; E-mail:
{drzhang.chao, hyf, cfli}@ustc.edu.cn, physics@alexpozas.com, armin.tavakoli@teorfys.lu.se

Supplementary Note 1 – Experimental data

Below we collect the experimental counts of coincidence events in the experiment, upon successful measurement of $b = 0$ in the central node.

$x_1 = 0, x_2 = 0, x_3 = 0$

a_1	a_2	a_3	coincidence counts
0	0	0	1481
0	0	1	3821
0	1	0	3663
0	1	1	708
1	0	0	3816
1	0	1	737
1	1	0	671
1	1	1	4633
total counts			19530

$x_1 = 0, x_2 = 0, x_3 = 1$

a_1	a_2	a_3	coincidence counts
0	0	0	720
0	0	1	4666
0	1	0	2632
0	1	1	1828
1	0	0	2855
1	0	1	1800
1	1	0	2921
1	1	1	2515
total counts			19937

$x_1 = 0, x_2 = 1, x_3 = 0$

a_1	a_2	a_3	coincidence counts
0	0	0	660
0	0	1	2614
0	1	0	4257
0	1	1	1706
1	0	0	2685
1	0	1	3064
1	1	0	1747
1	1	1	2163
total counts			18896

$x_1 = 0, x_2 = 1, x_3 = 1$

a_1	a_2	a_3	coincidence counts
0	0	0	2396
0	0	1	872
0	1	0	811
0	1	1	5426
1	0	0	5280
1	0	1	345
1	1	0	277
1	1	1	3735
total counts			19142

$x_1 = 1, x_2 = 0, x_3 = 0$

a_1	a_2	a_3	coincidence counts
0	0	0	709
0	0	1	2791
0	1	0	2282
0	1	1	2853
1	0	0	4420
1	0	1	1616
1	1	0	1778
1	1	1	2274
total counts			18723

$x_1 = 1, x_2 = 0, x_3 = 1$

a_1	a_2	a_3	coincidence counts
0	0	0	2559
0	0	1	839
0	1	0	5027
0	1	1	271
1	0	0	850
1	0	1	5812
1	1	0	326
1	1	1	4022
total counts			19706

$x_1 = 1, x_2 = 1, x_3 = 0$

a_1	a_2	a_3	coincidence counts
0	0	0	2311
0	0	1	5354
0	1	0	812
0	1	1	311
1	0	0	844
1	0	1	322
1	1	0	5440
1	1	1	3559
total counts			18953

$x_1 = 1, x_2 = 1, x_3 = 1$

a_1	a_2	a_3	coincidence counts
0	0	0	7064
0	0	1	680
0	1	0	625
0	1	1	508
1	0	0	714
1	0	1	562
1	1	0	464
1	1	1	9515
total counts			20132

Supplementary Note 2 – Experimental source independence

In order to estimate the degree of independence between the sources in the experiment, we calculate the mutual information (MI) between each pair of wing parties with different inputs $I(A^{(i)}; A^{(j)}) = H(A^{(i)}) + H(A^{(j)}) - H(A^{(i)}, A^{(j)})$, where $H(A^{(i)}) = -\sum_{a_i} p(a_i|x_i)\log p(a_i|x_i)$ is the Shannon entropy. For completely statistically independent parties, the mutual information should be zero. In the experiment, we set the three HWPs in the central node to 0° in order to project the three photons into the computational basis, and the three wing parties are switched between the A_0 and A_1 measurements randomly. By summing over all the outcomes of the central node, we obtain the joint and individual outcome probabilities of the wing parties. The calculated mutual information for different inputs is shown in Supplementary Table 1.

Supplementary Note 3 – FNN in the bilocal scenario

By removing one of the sources and one of the PBSs in the central party, we also test the full network nonlocality witnesses for the bilocal scenario. The experimental setup is shown in Supplementary Figure 1, where two sources are used to link the three nodes. As in the three-star case, Alice and Charlie both can perform two binary-outcome measurements, i.e., $x, z, a, c \in \{0, 1\}$. However, now the central party Bob performs a fixed measurement with three possible outcomes, so $b \in \{0, 1, 2\}$. In this scenario, all non-FNN

Supplementary Table 1: **The mutual information between pairs of parties.** Values close to zero verify the independence of the respective sources. Each row corresponds to a fixed choice of measurements for the pair of parties.

MI / $x_i x_j$	$I(A^{(1)}; A^{(2)})$	$I(A^{(1)}; A^{(3)})$	$I(A^{(2)}; A^{(3)})$
00	2.28e-04 ± 4.03e-04	5.99e-05 ± 2.65e-04	1.00e-03 ± 7.70e-04
01	3.38e-04 ± 4.76e-04	1.55e-04 ± 3.49e-04	3.46e-04 ± 4.96e-04
10	3.44e-06 ± 1.90e-04	2.79e-04 ± 4.44e-04	7.66e-05 ± 2.86e-04
11	1.57e-04 ± 3.57e-04	3.45e-04 ± 4.99e-04	2.43e-05 ± 2.28e-04

distributions $p(a, b, c|x, z)$ satisfy at least one of

$$\mathcal{R}_{\text{C-NS}} = 2\langle A_0 B_1 C_0\rangle - 2\langle A_0 B_1 C_1\rangle + 2\langle A_1 B_0 C_0\rangle + \langle A_1 B_0 C_1\rangle \\ -\langle B_0\rangle + [\langle A_1 B_0\rangle + \langle B_0 C_0\rangle - \langle C_0\rangle]\,\langle C_1\rangle \leq 3 \tag{1}$$

and

$$\mathcal{R}_{\text{NS-C}} = 2\langle A_0 B_1 C_0\rangle - 2\langle A_0 B_1 C_1\rangle + \langle A_1 B_0 C_0\rangle + 2\langle A_1 B_0 C_1\rangle \\ -\langle B_0\rangle + \langle A_1\rangle\,[\langle A_1 B_0\rangle + \langle B_0 C_1\rangle + \langle C_0\rangle - \langle C_1\rangle - \langle A_1\rangle] \leq 3, \tag{2}$$

where we define $\langle A_x B_0 C_z\rangle = \sum_{a,b,c}(-1)^{a+c}[p(a, 0, c|x, z) + p(a, 1, c|x, z) - p(a, 2, c|x, z)]$ and $\langle A_x B_1 C_z\rangle = \sum_{a,b,c}(-1)^{a+c}[p(a, 0, c|x, z) - p(a, 1, c|x, z)]$.

In the experiment, both SPDC sources produce entangled photon pairs in the singlet state $\frac{1}{\sqrt{2}}(|HV\rangle - |VH\rangle)$. The measurement observables for Alice and Charlie are $A_0 = \sigma_x$, $A_1 = \sigma_z$, $C_0 = \frac{\sigma_z+\sigma_x}{\sqrt{2}}$ and $C_1 = \frac{\sigma_z-\sigma_x}{\sqrt{2}}$. At the central node, Bob performs a partial Bell state measurement by overlapping the two input photons on a PBS and detected in the $|\pm\rangle$ basis. In order to detect three outcomes, Bob implements pseudo-number-resolving detectors which consist of a 22.5° HWP, a PBS and two fiber-coupled avalanche photodiodes (APDs) for each of his four output ports. Thus the partial BSM can resolve the two states $|\phi^{\pm}\rangle = (|HH\rangle + |VV\rangle)/\sqrt{2}$, and the unresolved events are associated to the outcome $b = 2$. The resulting theoretical distribution leads to the quantum violations $\mathcal{R}_{\text{C-NS}} = \mathcal{R}_{\text{NS-C}} = 5/\sqrt{2} \approx 3.5355$.

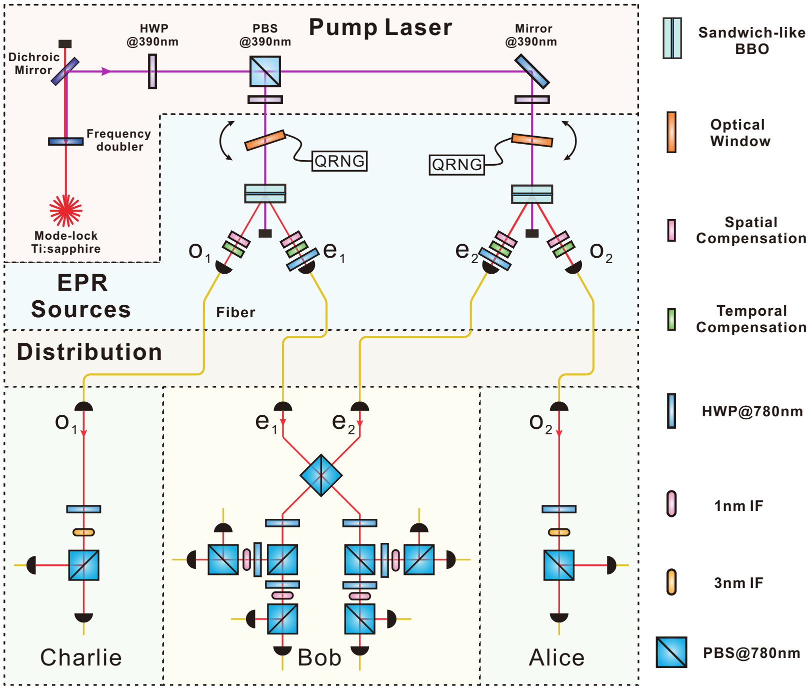

Supplementary Figure 1: **Experimental setup for the FNN experiment in the bilocal scenario.** The ultraviolet pulse is divided into two parallel beams and the relative phases between them are erased by two randomly rotated optical windows. Ordinary photons from two EPR sources are sent to the two end nodes respectively. Extraordinary photons are sent to the central node and measured by a partial Bell state measurement device. PBS, polarisation beam splitter; HWP, half-wave plate; IF, interference filter; QRNG, quantum random number generator; BBO, beta barium borate.

We use 1- and 3-nm bandwidth filters for, respectively, the e- and o-polarised photons, and use a pump power of 30 mW for each source. The two-photon counting rate is about 9000 Hz and the four-photon coincidence rate is about 0.8 Hz. As in the experiment described in the main text, in order to improve the independence of the sources we insert a randomly rotated glass slice before each source. After collecting data for 10 000 seconds for each setting, the experimentally measured results are $\mathcal{R}_{\text{C-NS}} = 3.4966 \pm 0.0238$ and $\mathcal{R}_{\text{NS-C}} = 3.4166 \pm 0.0237$, violating the non-FNN bounds by more than 17 standard deviations. The raw data is shown below. We note that the raw data must be post-processed for the events with $b = 2$ due to the limited photon-resolving capabilities of the setup. Namely, when the input to Bob's measurement station is $|\psi^{\pm}\rangle = (|01\rangle \pm |10\rangle)/\sqrt{2}$, the output from the top PBS is two indistinguishable photons either in the left part or

in the right part of the station. These two photons have a 50% chance of arriving to different output ports (i.e., producing a two-click event), and 50% chance of arriving to the same port (and producing just one click). The raw data in the tables below only contains 4-click events in the whole experiment, thus effectively reducing the $b = 2$ events in half. In order to correct for this, we multiply by 2 the counts of $b = 2$ events in the raw data and compute $p(a, b, c|x, z)$ according to the post-processed coincidence counts.

$x = 0, z = 0$

a	b	c	coincidence counts
0	0	0	1053
0	0	1	255
0	1	0	224
0	1	1	1193
0	2	0	655
0	2	1	749
1	0	0	233
1	0	1	1134
1	1	0	1000
1	1	1	247
1	2	0	642
1	2	1	702
total counts			8087

$x = 0, z = 1$

a	b	c	coincidence counts
0	0	0	208
0	0	1	1255
0	1	0	1155
0	1	1	215
0	2	0	630
0	2	1	737
1	0	0	1090
1	0	1	190
1	1	0	215
1	1	1	1104
1	2	0	600
1	2	1	691
total counts			8090

$x = 1, z = 0$

a	b	c	coincidence counts
0	0	0	1105
0	0	1	180
0	1	0	1163
0	1	1	195
0	2	0	167
0	2	1	1297
1	0	0	159
1	0	1	1254
1	1	0	174
1	1	1	1179
1	2	0	1067
1	2	1	160
total counts			8100

$x = 1, z = 1$

a	b	c	coincidence counts
0	0	0	1017
0	0	1	241
0	1	0	1111
0	1	1	204
0	2	0	212
0	2	1	1160
1	0	0	186
1	0	1	1110
1	1	0	229
1	1	1	1161
1	2	0	996
1	2	1	221
total counts			7848
